# Identification of Potential Diagnostic and Prognostic Biomarkers for Gastric Cancer Based on Bioinformatic Analysis

**DOI:** 10.3389/fgene.2022.862105

**Published:** 2022-03-16

**Authors:** Xiaoji Niu, Liman Ren, Aiyan Hu, Shuhui Zhang, Hongjun Qi

**Affiliations:** ^1^ Department of Gastroenterology of Traditional Chinese Medicine, Qinghai Province Hospital of Traditional Chinese Medicine, Xining, China; ^2^ Department of Pathology, Yueyang Hospital of Integrated Traditional Chinese and Western Medicine, Shanghai University of Traditional Chinese Medicine, Shanghai, China; ^3^ Department of Endocrinology, Qinghai Province Hospital of Traditional Chinese Medicine, Xining, China

**Keywords:** gastric cancer, bioinformatics analysis, microarray, differentially expressed genes, prognosis, diagnosis

## Abstract

**Background:** Gastric cancer (GC) is one of the most prevalent cancers all over the world. The molecular mechanisms of GC remain unclear and not well understood. GC cases are majorly diagnosed at the late stage, resulting in a poor prognosis. Advances in molecular biology techniques allow us to get a better understanding of precise molecular mechanisms and enable us to identify the key genes in the carcinogenesis and progression of GC.

**Methods:** The present study used datasets from the GEO database to screen differentially expressed genes (DEGs) between GC and normal gastric tissues. GO and KEGG enrichments were utilized to analyze the function of DEGs. The STRING database and Cytoscape software were applied to generate protein–protein network and find hub genes. The expression levels of hub genes were evaluated using data from the TCGA database. Survival analysis was conducted to evaluate the prognostic value of hub genes. The GEPIA database was involved to correlate key gene expressions with the pathological stage. Also, ROC curves were constructed to assess the diagnostic value of key genes.

**Results:** A total of 607 DEGs were identified using three GEO datasets. GO analysis showed that the DEGs were mainly enriched in extracellular structure and matrix organization, collagen fibril organization, extracellular matrix (ECM), and integrin binding. KEGG enrichment was mainly enriched in protein digestion and absorption, ECM-receptor interaction, and focal adhesion. Fifteen genes were identified as hub genes, one of which was excluded for no significant expression between tumor and normal tissues. COL1A1, COL5A2, P4HA3, and SPARC showed high values in prognosis and diagnosis of GC.

**Conclusion:** We suggest COL1A1, COL5A2, P4HA3, and SPARC as biomarkers for the diagnosis and prognosis of GC.

## Introduction

According to data published in 2021, gastric cancer (GC), among all cancers, ranked fourth in cancer-related deaths (Sung et al., 2021). Stomach adenocarcinoma (STAD), the most common histological type, accounts for more than 90% of GC (Ajani et al., 2017). Although endoscopy or histological detection has developed a lot in recent years, the majority of GC patients are diagnosed at their late and advanced stage due to an insidious onset, resulting in high morbidity and mortality (Chen et al., 2020; Wang W. et al., 2020). However, advances in molecular biology techniques allow us to approach precise molecular mechanisms of carcinogenesis and enable us to find potential diagnostic and prognostic biomarkers for GC.

Previous bioinformatic studies resulted in different biomarkers due to different screening criteria and different datasets from Gene Expression Omnibus (GEO) (Sun et al., 2017; Zheng H.-C. et al., 2017; Shi and Zhang, 2019). In the present study, we identified DEGs based on three datasets from GEO, GSE19826, GSE54129, and GSE118916. Gene Ontology (GO) and Kyoto Encyclopedia of Genes and Genomes (KEGG) pathway enrichment analysis were performed subsequently. Afterwards, we constructed the protein–protein interaction (PPI) network to identify hub genes using the STRING database and Cytoscape software. Then, we performed the survival analysis, including overall survival (OS), disease-free survival (DSS), and progress-free interval (PFI) to identify candidate genes. The expression of candidate genes and their correlation with the pathological stage were further analyzed along with the diagnostic value. A total of four genes were identified as potential biomarkers for GC in our study.

## Materials and Methods

### Microarray Datasets

RNA-sequencing datasets containing gastric cancer tissue samples and normal tissue samples were obtained from the GEO database (Barrett et al., 2013), (https://www.ncbi.nlm.nih.gov/geo/) and three GEO datasets, including GSE19826 (Wang Q. et al., 2012), GSE54129, and GSE118916 (Li et al., 2019), were downloaded for further analysis.

### Identification of Differentially Expressed Genes

The limma package (version: 3.40.2) of R software was used to identity the DEGs in three datasets. The adjusted *p* value was analyzed to correct for false positive results in GEO datasets. “Adjusted *p* < 0.05 and fold change >1.5” were defined as the thresholds for the screening of the differential expression of mRNAs. Subsequently, the ggplot package (version: 3.3.3) of R software was used to make a Venn diagram to extract the common DEGs of the three datasets.

### Enrichment Analysis of Differentially Expressed Genes

The clusterProfiler package (Yu et al., 2012) (version 3.14.3) of R software was used for enrichment analysis with the following ontology sources: GO biological processes (BPs), cellular components (CCs), molecular functions (MFs), and KEGG pathway. Adjusted *p* < 0.05 and q < 0.2 were set as the critical standard for significant enrichment.

### Analysis of Protein–Protein Interaction Network

The PPI network of DEGs was generated using the search tool of the STRING database (Szklarczyk et al., 2019) (version 11.5). The “Multiple Proteins by Names/Identifiers” tool was chosen in this study. The organism was set as “*Homo Sapiens*.” Required score was set as high confidence (0.700), and FDR stringency was set to medium (5%). The PPI network was exported for further analysis with the Cytoscape software (Otasek et al., 2019) (version 3.8.2). The plugin MCODE (Bandettini et al., 2012) (version 2.0.0, degree cutoff: 2, node score cutoff: 0.2, K-score: 2) was applied to identify the hub genes in the PPI network. The module with the highest degree was used in the following analysis.

### The Expressions and Survival Analysis of Hub Genes

The Cancer Genome Atlas (TCGA) project is an open database aiming to link cancer genomic data to patients’ clinicopathological information (https://www.cancer.gov/tcga). Raw counts of RNA-sequencing data (level 3) were obtained from TCGA along with corresponding clinicopathological information (Liu et al., 2018). TPM-formatted RNA-sequencing data of normal tissues from the Genotype-Tissue Expression Project (GTEx) were obtained from the University of California Santa Cruz (Vivian et al., 2017) (https://xenabrowser.net/datapages/). Tumor/normal differential expression analyses of hub genes were conducted using R software. We conducted the survival analysis, including the OS, DSS, and PFI, with the Xiantao Academic platform (survminer package of R software). DEGs related to the OS, DSS, and PFI were considered as our purpose genes and were involved in the following data analysis.

### Correlation Analysis of Purpose Genes

GEPIA (Tang et al., 2017) (http://gepia.cancer-pku.cn/) is a database that enable users to analyze the RNA-sequencing expression in various ways. We used GEPIA to correlate our purpose genes with the pathological stage. Correlation analysis among purpose genes were conducted using R software embedded in Xiantao Academic. Correlation among these purpose genes were visualized with a heat map generated by the ggplot package.

### Statistical Analysis

Xiantao Academic (https://www.xiantao.love/products) is a platform embedded with R software and R packages for data analyzing. The major analysis was performed using Xiantao Academic in the present study. Chi-square test and the Wilcoxon rank sum test were utilized in the analysis depending on the data. Spearman correlation analysis was used in different expression of genes. In the analysis of the correlation of gene expression with pathological stage, the expression data are first log2 (TPM+1) transformed, and the method was one-way ANOVA, using pathological stage as a variable for calculating differential expression. *p* value <0.05 was regarded as statistically significant.

## Results

### Identification of Differentially Expressed Genes

**FIGURE 1:**
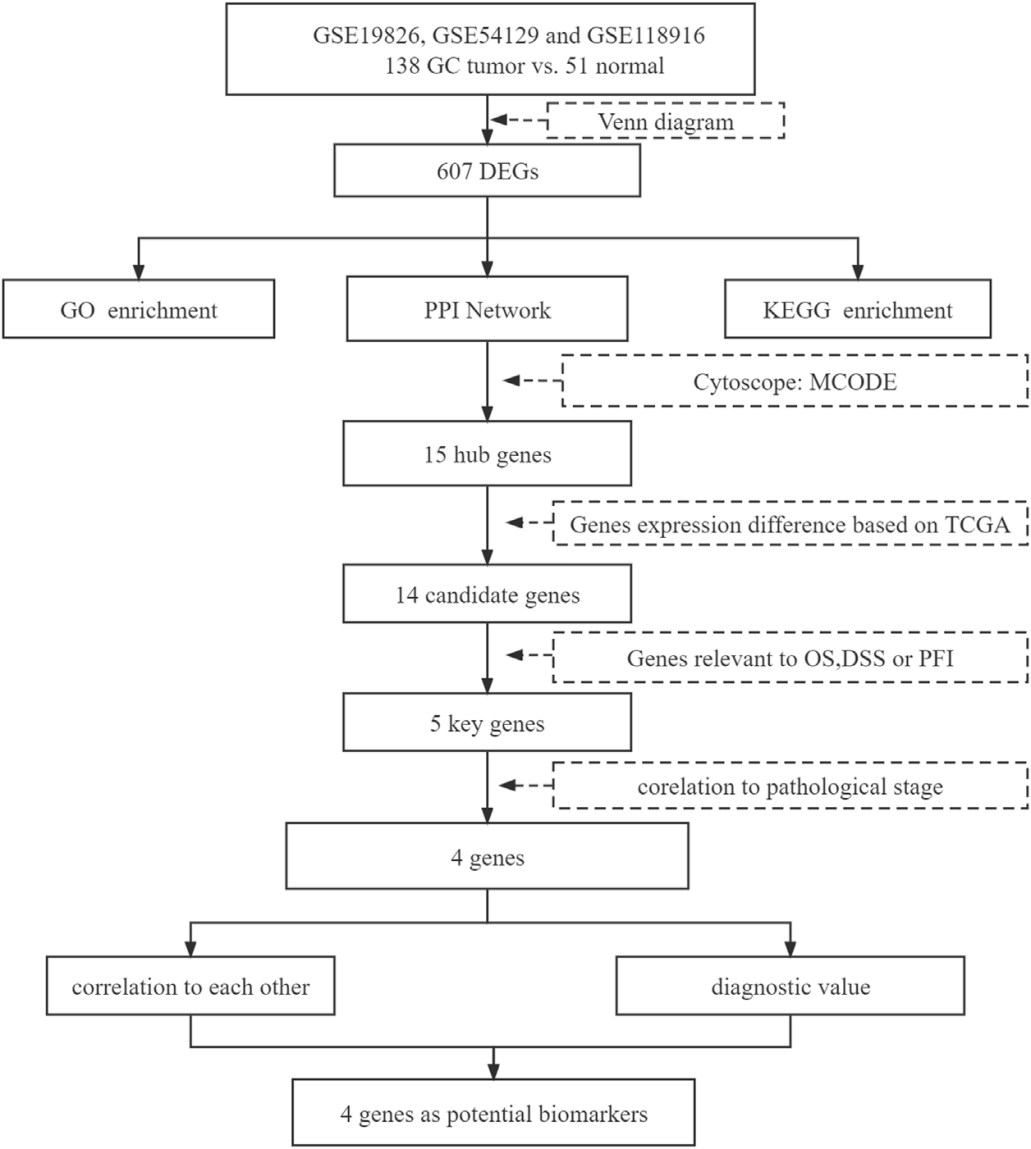
Flowchart diagram for bioinformatics analysis.

**FIGURE 2:**
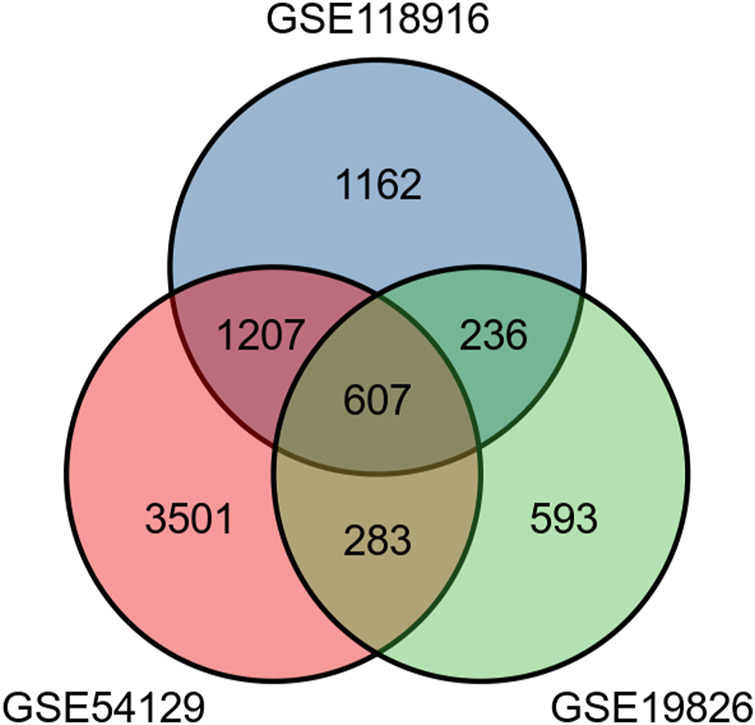
The Venn diagram shows a total of 607 differentially expressed genes including 294 up-regulated genes and 313 down-regulated genes.

The present study involved three GEO datasets, GSE19826, GSE54129, and GSE118916. GSE19826 contained 12 pairs of samples from GC tumor and adjacent non-tumor tissues and three normal tissues. GSE54129 contained 111 GC tumor tissue samples and 21 normal tissue samples. GSE118916 contained 15 pairs of Gastric cancer tumor and adjacent non-tumor (normal) tissues. There were 138 GC tumor tissue samples and 51 normal tissue samples in total involved in the present study. The flow chart is shown in Figure 1. We identified 607 DEGs including 294 up-regulated genes and 313 down-regulated genes in GC tissue samples (Figure 2).

### Functional Enrichment Analysis of Differentially Expressed Genes

**FIGURE 3:**
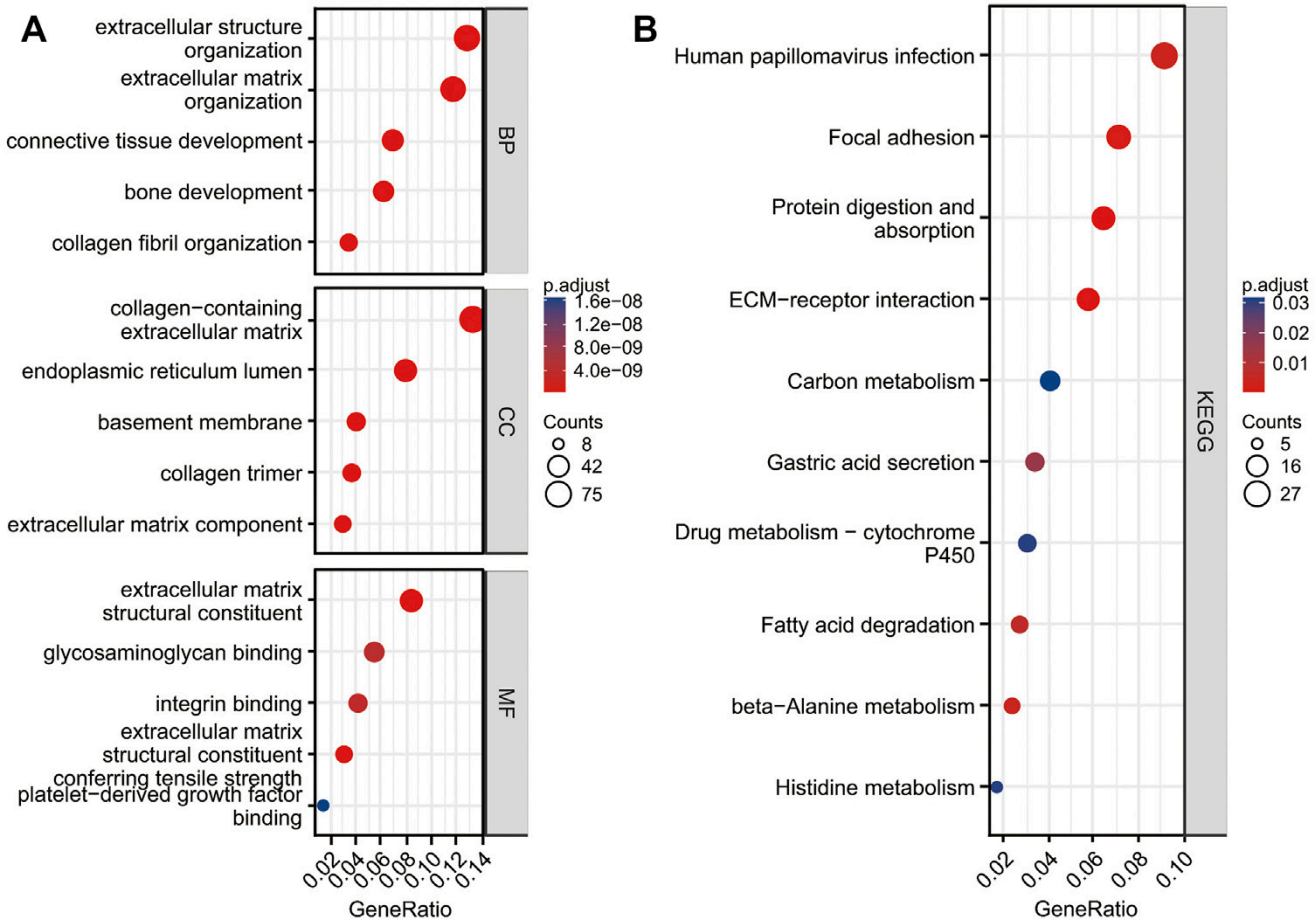
Functional analysis of DEGs. Top five GO terms enrichment in biological process (BP), cell composition (CC), and molecular function (MF) **(A)**. KEGG enrichment of DGEs **(B)**.

We conducted a functional enrichment analysis of DEGs using R software and R codes embedded in Xiantao platform. DEGs are enriched in 341 terms of GO BP, including extracellular structure organization, extracellular matrix (ECM) organization, collagen fibril organization, bone development and connective tissue development, etc. DEGs were enriched in 43 terms of GO CC, including collagen-containing extracellular matrix, endoplasmic reticulum lumen, basement membrane, extracellular matrix component and collagen trimer, etc. DEGs were enriched in 35 terms of GO MF, including extracellular matrix structural constituent, extracellular matrix structural constituent conferring tensile strength, integrin binding, glycosaminoglycan binding, and platelet-derived growth factor binding (Figure 3A; Supplementary Table S1). DEGs were enriched in 10 terms of KEGG, including protein digestion and absorption, ECM-receptor interaction, Focal adhesion, human papillomavirus infection, beta-alanine metabolism, fatty acid degradation, gastric acid secretion, histidine metabolism, drug metabolism—cytochrome P450, and carbon metabolism (Figure 3B; Supplementary Table S1).

### Protein–Protein Interaction Network to Identify Hub Genes

**FIGURE 4:**
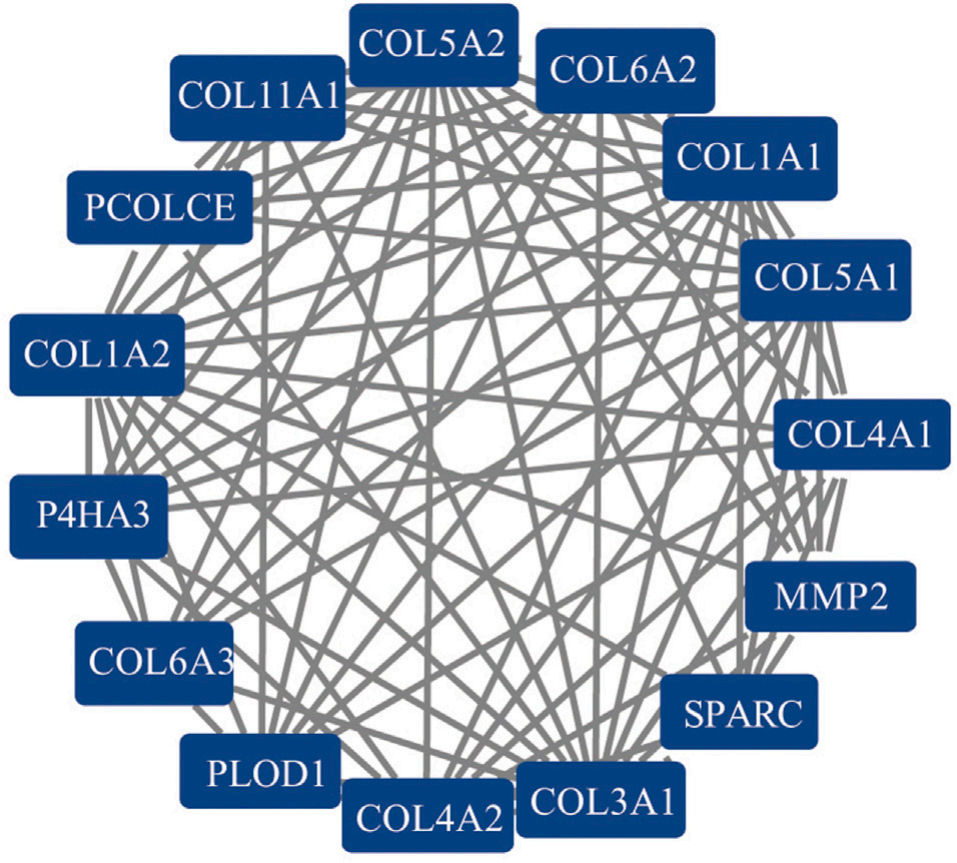
The PPI network of 15 hub genes selected with the MCODE plugin of Cytoscape.

**FIGURE 5:**
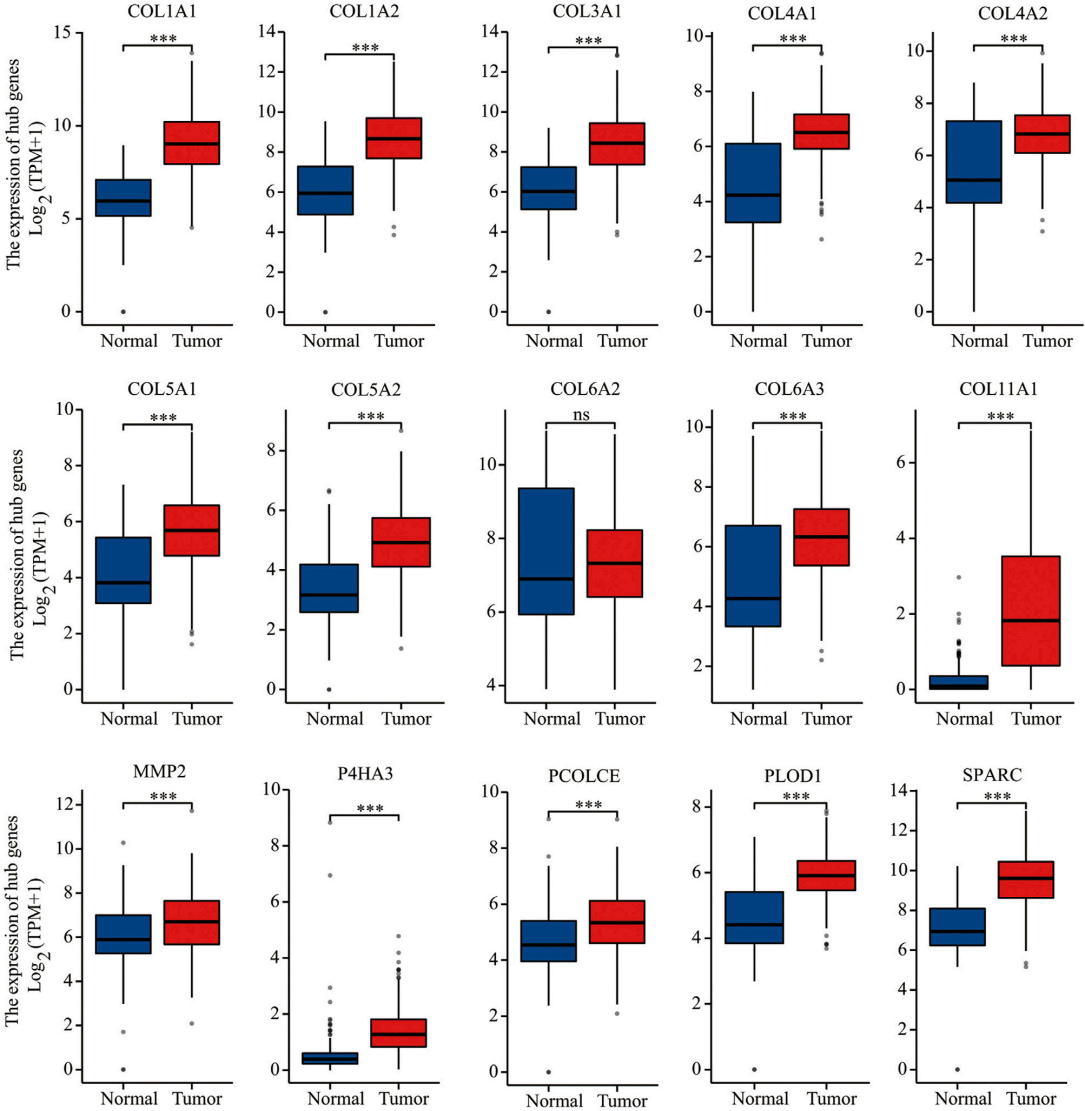
Gene expression of 15 hub genes (COL1A1, COL1A2, COL3A1, COL4A1, COL4A2, COL5A1, COL5A2, COL6A2, COL6A3, COL11A1, MMP2, P4HA3, PCOLCE, PLOD1, and SPARC) based on TGCA and GTEx databases. ***p < 0.001; ns, not statistically significant.

A PPI network of 607 DEGs, containing a total of 317 nodes and 606 edges, was generated using STRING, and an interaction score >0.7 was considered a high-confidence interaction relationship. We identified 15 nodes and 81 edges with MCODE plugin. The module with the highest degree was used in the following analysis. The hub genes included COL1A1, COL1A2, COL3A1, COL4A1, COL4A2, COL5A1, COL5A2, COL6A2, COL6A3, COL11A1, MMP2, P4HA3, PCOLCE, PLOD1, and SPARC (Figure 4). Gene expression profiles of the 15 hub genes between GC tumor samples and normal samples are shown in Figure 5. The expression of COL6A2 showed no difference in tumor and normal tissues, so it was excluded in further analyses. The remaining 14 genes were considered as candidate genes for potential diagnostic and prognostic biomarkers.

### Survival and Correlation Analysis

**FIGURE 6:**
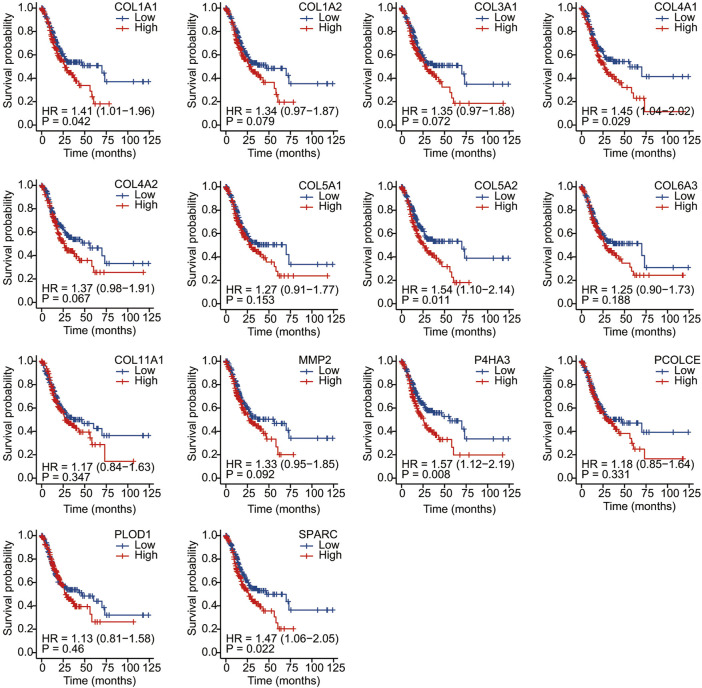
Overall survival analysis of 14 candidate genes (COL1A1, COL1A2, COL3A1, COL4A1, COL4A2, COL5A1, COL5A2, COL6A3, COL11A1, MMP2, P4HA3, PCOLCE, PLOD1, and SPARC). COL1A1, COL4A1, COL5A2, P4HA3, and SPARC (HR = 1.47, *p* = 0.022) were associated with OS.

**FIGURE 7:**
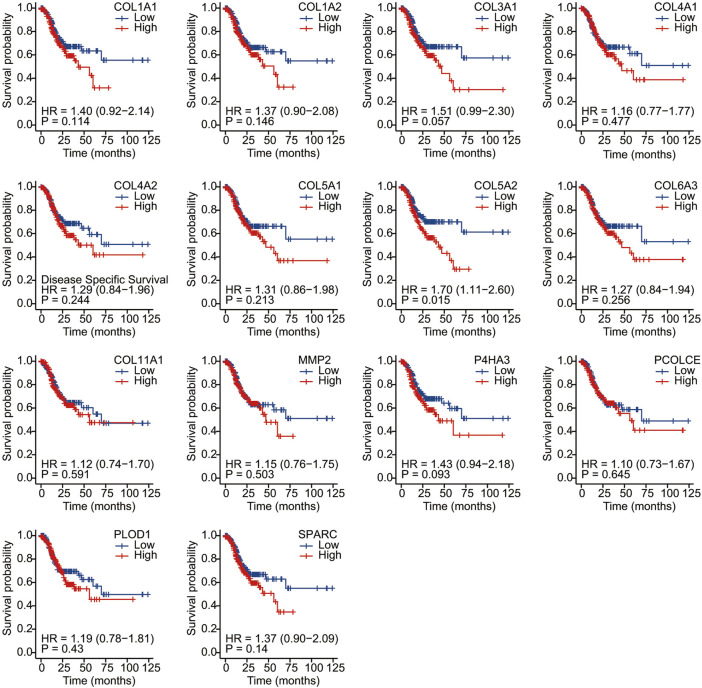
Disease-specific survival analysis of 14 candidate genes (COL1A1, COL1A2, COL3A1, COL4A1, COL4A2, COL5A1, COL5A2, COL6A3, COL11A1, MMP2, P4HA3, PCOLCE, PLOD1, and SPARC). COL5A2 was associated with DSS (HR = 1.70, *p* = 0.015).

**FIGURE 8:**
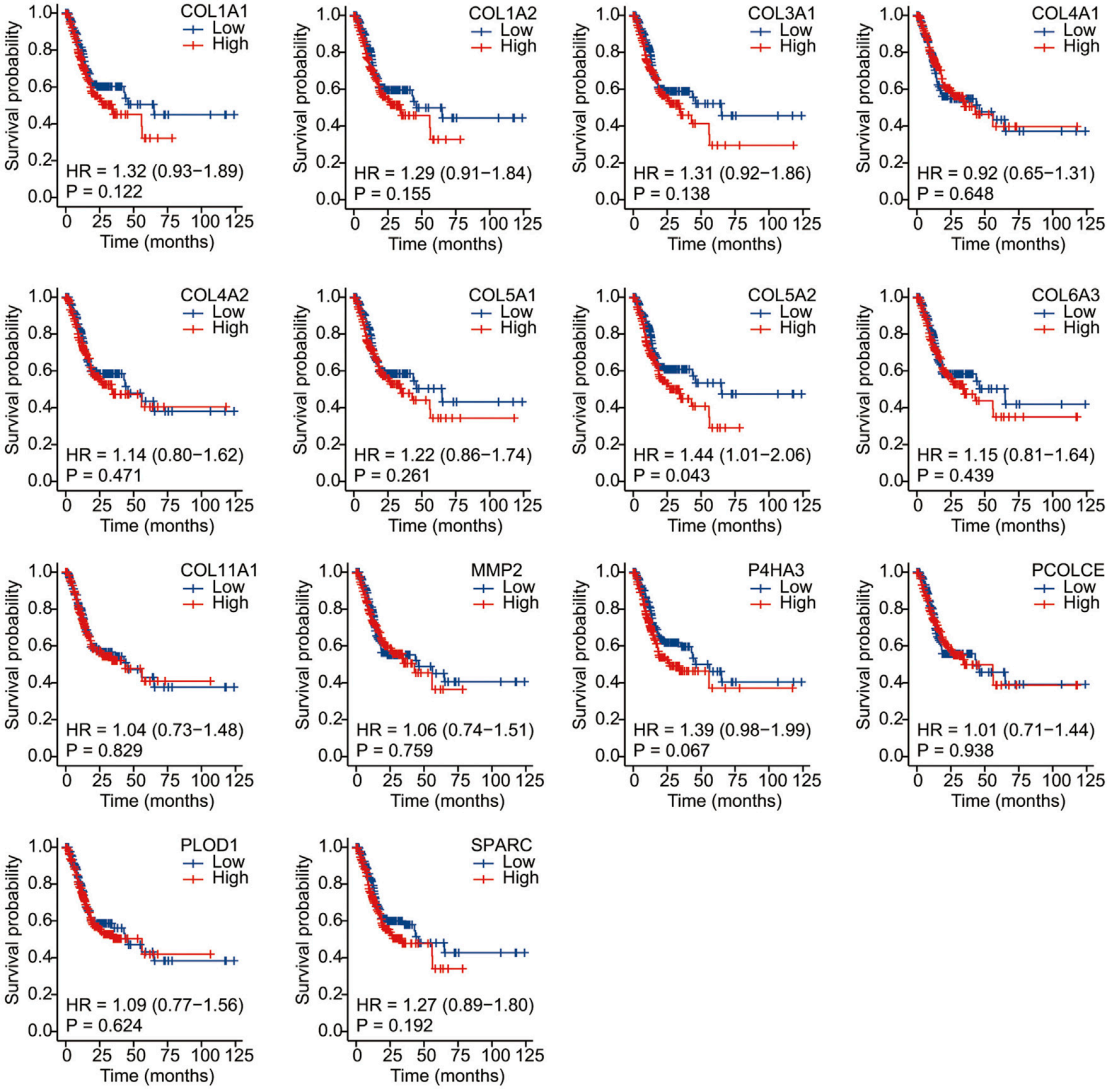
Progress free interval analysis of 14 candidate genes (COL1A1, COL1A2, COL3A1, COL4A1, COL4A2, COL5A1, COL5A2, COL6A3, COL11A1, MMP2, P4HA3, PCOLCE, PLOD1, and SPARC). COL5A2 was associated with PFI (HR = 1.44, *p* = 0.043).

**FIGURE 9:**
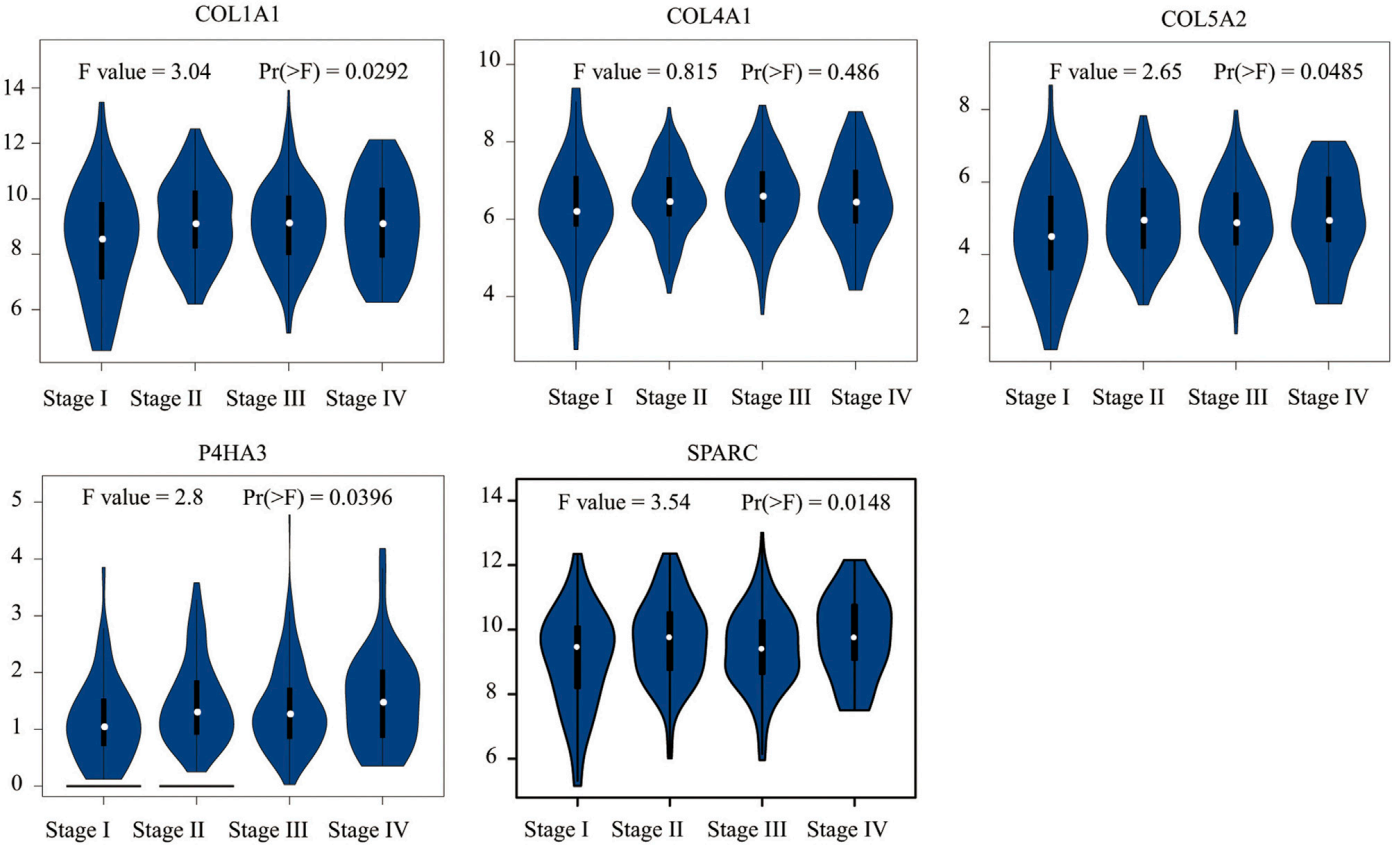
Correlation analysis between five key genes (COL1A1, COL4A1, COL5A2, P4HA3, and SPARC) and the pathological stage of GC shows they are potential prognostic markers.

We conducted Kaplan–Meier survival analysis with the candidate genes. Candidate genes related to OS, DSS, or PFI were considered as key genes. Among the 14 candidate genes, COL1A1 (HR = 1.41, *p* = 0.042), COL4A1 (HR = 1.45, *p* = 0.029), COL5A2 (HR = 1.54, *p* = 0.011), P4HA3 (HR = 1.57, *p* = 0.011), and SPARC (HR = 1.47, *p* = 0.022) were associated with the OS of STAD (Figure 6). COL5A2 was associated with DSS (HR = 1.70, *p* = 0.015) (Figure 7) and PFI (HR = 1.44, *p* = 0.043) (Figure 8). Therefore, this study focused on the five key genes, COL1A1, COL4A1, COL5A2, P4HA3, and SPARC. Further analysis of the correlation between these key genes and the pathological stage of GC showed that COL1A1, COL5A2, P4HA3, and SPARC were significantly correlated to cancer pathological stages. However, COL4A1 showed no significance in the correlation analysis (Figure 9). Therefore, we identify COL1A1, COL5A2, P4HA3, and SPARC as potential biomarkers for prognosis of GC.

### Correlation Expression and Diagnostic Analysis

**FIGURE 10:**
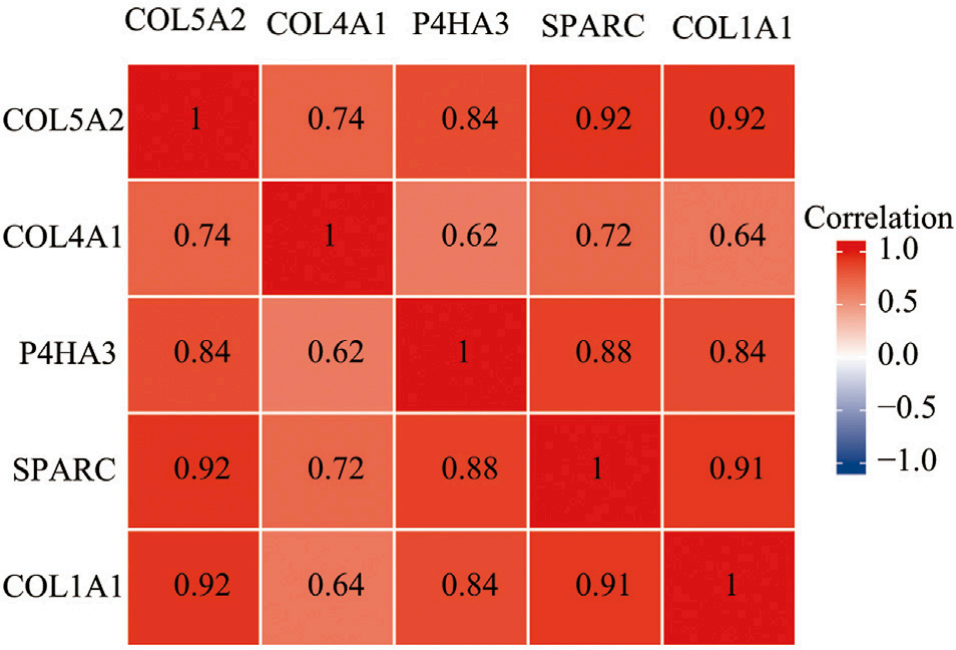
The expression of four genes (COL1A1, COL5A2, P4HA3, and SPARC) are correlated with each other in GC.

**FIGURE 11:**
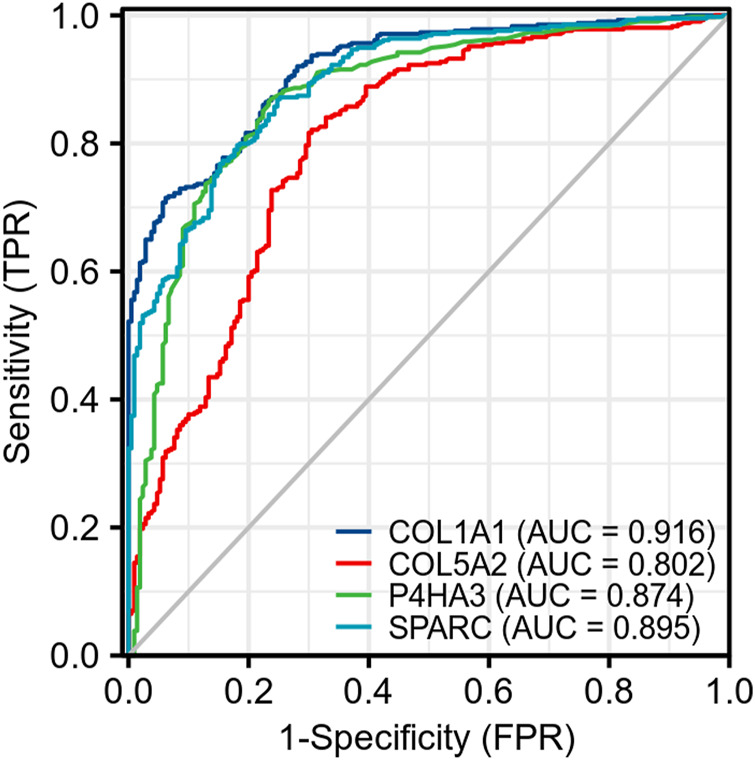
ROC of four key genes (COL1A1, COL5A2, P4HA3, and SPARC) shows they are of high diagnostic value in GC.

We analyzed the correlation between these four genes on Xiantao Academic based on data from TCGA and found that all of these genes were highly correlated with each other. The r value ranged from 0.84 to 0.92 (*p* < 0.01) (Figure 10). We used a receiver operating characteristic (ROC) curve to assess the diagnostic value of the purpose genes using Xiantao Academic tools based on TCGA and GTEx samples. The area under curve (AUC) of COL1A1, COL5A2, P4HA3, and SPARC was 0.916, 0.802, 0.874, and 0.895, respectively. The results, as shown previously, suggested that these four genes we selected could effectively distinguish GC samples with normal samples (Figure 11). COL1A1, COL5A2, P4HA3, and SPARC could be biomarkers for the diagnosis and prognosis of GC.

## Discussion

GC is one of the most diagnosed cancers and has brought great burden to global health. Patients were likely to be diagnosed in their late stage due to the lack of specific clinical symptoms at an early stage. Thus, patients with GC have poor prognosis. It is urgent to identify relevant biomarkers that are valid for both diagnostic and prognostic evaluation. Bioinformatics analysis enables us to explore the genetic alterations in GC and has been proved to be a useful approach to identify new biomarkers in plenty of diseases. An initial objective of the project was to identify appropriate biomarkers of GC using bioinformatics analysis.

In the current study, we identified 607 DEGs meeting the criteria. GO enrichment suggested those genes were significantly associated with extracellular structure and matrix organization, collagen fibril organization, and ECM and integrin binding. KEGG was mainly enriched in protein digestion and absorption, ECM-receptor interaction, and focal adhesion. In accordance with the present results, previous studies have reported that cancer-associated fibroblasts are essential in creating extracellular matrix structure and metabolism and account for the adaptive resistance to chemotherapy caused by immune reprogramming of the tumor microenvironment (Quante et al., 2011; Kalluri, 2016). Extracellular matrix plays a significant part in the creation of tumor microenvironment and promotes malignancy (Madsen and Sidenius, 2008; Najafi et al., 2019; Mohan et al., 2020; Piersma et al., 2020; Wang W. et al., 2020). Integrins coordinate ECM–cell and cell–cell interactions, signal transmission, gene expression, and cell function. The interaction between integrin and the cancer glycol microenvironment plays a significant part in regulating cancer progression (Marsico et al., 2018).

The results of this study showed that COL1A1, COL4A1, COL5A2, P4HA3, and SPARC were associated with the OS of GC. COL5A2 was associated with DSS and PFI. Further analysis of the correlation between these key genes and the pathological stages of GC showed that COL4A1 showed no significance in the correlation analysis to pathological stages. Therefore, we identify four genes as potential biomarkers of GC including COL1A1, COL5A2, P4HA3, and SPARC. Also, the diagnostic value of these genes was confirmed in the following analysis.

COL1A1 is an important member of the type-I collagen family, the main fibrillar collagen and an essential structural component of the ECM (Li J. et al., 2016). Many bioinformatic analyses identified COL1A1 as a biomarker of GC (Wang W. et al., 2020; Wang Y. et al., 2021; Zhao et al., 2021). Abnormal expression of COL1A1 has been reported in several cancers, including hepatocellular carcinoma, ovarian cancer, and colorectal cancer, as well as in GC (Li J. et al., 2016; Zhang et al., 2018; Ma et al., 2019; Li et al., 2020). *In vitro*, enhanced expression of COL1A1 promotes the invasion and migration of GC cells, while knocking out COL1A1 inhibits the increase in cell metastasis ability (Li et al., 2021). It plays an important role in promoting tumor cell proliferation, migration, invasion, epithelial–mesenchymal transformation (EMT), and chemotherapy resistance (Armstrong et al., 2004; Koenig et al., 2006; Shintani et al., 2008; Yang et al., 2014; Zheng X. et al., 2017; Yamazaki et al., 2018; Shi et al., 2021). ROC analysis showed high diagnostic value of COL1A1 (AUC = 0.916) based on 414 GC samples and 210 normal gastric tissues. This finding is consistent with that of Zhao et al. (2021) (AUC = 0.917) based on 375 GC samples and 32 normal samples (Zhao et al., 2021). The diagnostic and prognostic values of COL1A1 were confirmed with extra data (more samples than others) from the present work.

COL5A2 is a member of the type-V collagen family which is also a significant structural component of the ECM. COL5A2 was reported to promote proliferation and invasion in colon cancer and prostate cancer (Ren et al., 2021; Wang J. et al., 2021). Also, it has a strong correlation to the prognosis of renal cancer and gastric cancer (Ding et al., 2021; Tan et al., 2021). The overexpression of COL5A2 promoted the migration of GC cells *in vitro* and *in vivo*, and the knockdown of COL5A2 could significantly decrease the migration of cell (Tan et al., 2021). A previous study had demonstrated that patients with higher COL5A2 levels were more likely to suffer from renal metastasis (AUC = 0.878). Among all those genes we identified as potential biomarkers, COL5A2 was the unique gene that was associated with the OS, DSS, and PFI of GC, which had not been reported in previous studies. The value of AUC in our current study is 0.802 based on data from TCGA and GTEx. Therefore, COL5A2 could serve as a novel biomarker of GC. Also, we would perform biological experiments to verify the result.

Previous research showed that P4HA3 was up-regulated in head and neck squamous cell carcinoma (HNSCC) tissue, and it was demonstrated to promote HNSCC cell proliferation, invasion, and migration *in vitro* (Wang T. et al., 2020). A recent study showed that the de-regulation of P4HA3 was associated with increased metastasis and poor prognosis of GC (Song et al., 2018). In the present work, the value of AUC of P4HA3 is 0.875, which indicated high value of diagnosis and has not been reported in previous studies. The result suggests that P4HA3 is a potential biomarker of GC.

SPARC is one of the first-known matricellular protein that modulates interactions between cells and the ECM. It has divergent actions due to different categories of tumors. It shows anti-tumor or tumor-promoting effects in different cancers (Tai and Tang, 2008). What is surprising is that previous research results are inconsistent. As Zhang et al. (2012) and Zhang et al. (2014) reported, “SPARC expression is negatively correlated with the clinicopathological factors of gastric cancer and inhibits malignancy of gastric cancer cells,” and they confirmed the anti-tumor activity of SPARC *in vivo* and *in vitro*. The anti-tumor activity was also reported by Wang L. et al. (2012) in a clinical trial involving 80 gastric cancer samples and 30 normal samples. On the contrary, the tumor-promoting effect of SPARC was also reported in GC. Over expression of SPARC promoted GC progression, including serosal invasion, lymph node, and distant metastasis, and tended to poor prognosis of patients (Zhao et al., 2010; Sato et al., 2013; Wang et al., 2014). Also, the invasion and proliferation ability was inhibited in SPARC knockdown MGC803 and HGC 27 gastric cancer cell lines, which demonstrated the tumor-promoting activity of SPARC. Increased expression of SPARC in this study corroborates these earlier findings (Li Z. et al., 2016; Liao et al., 2018; Li et al., 2019). ROC analysis showed high diagnostic value of SPARC, and the value of AUC was 0.895 in the current study. Biological experiments in different cell lines and clinical samples are necessary to verify the result.

The correlation between these four genes was analyzed, and we found that all of these genes were highly correlated with each other, which enhanced their possibility as potential biomarkers of GC.

In summary, previous studies have identified COL1A1 as a biomarker for GC diagnosis and prognosis. COL5A2, P4HA3, and SPARC were reported to be associated with poor prognosis (OS and DSS, but not PFI); however, the diagnostic value has not been recognized. In the present study, the prognosis values of COL1A1, COL5A2, P4HA3, and SPARC were confirmed. The ROC analysis showed that they could distinguish between GC samples and normal samples effectively. Thus, we suggest COL1A1, COL5A2, P4HA3, and SPARC as biomarkers for both diagnosis and prognosis of GC. Each of the biomarkers identified in the present work plays a significant role in the ECM, which highlights the importance of the tumor microenvironment in GC. Compared with similar studies, we suggested those genes as both diagnostic and prognostic biomarkers for GC. Nevertheless, the current results are all derived from bioinformatics analysis and are limited by the absence of confirmation. Due to different screening criteria, previous bioinformatics research produced different biomarkers. Many of the biomarkers have been verified, and the combination of those results might be more rigorous. Further clinical experiments are underway to verify their value in GC.

## Data Availability

Publicly available datasets were analyzed in this study. These data can be found at https://www.ncbi.nlm.nih.gov/geo/download/?acc = GSE19826; https://www.ncbi.nlm.nih.gov/geo/download/?acc = GSE54129; and https://www.ncbi.nlm.nih.gov/geo/download/?acc = GSE118916.

## References

[B1] Ajani J. A. Lee J. Sano T. Janjigian Y. Y. Fan D. Song S. (2017). Gastric Adenocarcinoma. Nat. Rev. Dis. Primers 3 (1), 17036. 10.1038/nrdp.2017.36 28569272

[B2] Armstrong T. Packham G. Murphy L. B. Bateman A. C. Conti J. A. Fine D. R. (2004). Type I Collagen Promotes the Malignant Phenotype of Pancreatic Ductal Adenocarcinoma. Clin. Cancer Res. 10 (21), 7427–7437. 10.1158/1078-0432.CCR-03-0825 15534120

[B3] Bandettini W. P. Kellman P. Mancini C. Booker O. J. Vasu S. Leung S. W. (2012). MultiContrast Delayed Enhancement (MCODE) Improves Detection of Subendocardial Myocardial Infarction by Late Gadolinium Enhancement Cardiovascular Magnetic Resonance: A Clinical Validation Study. J. Cardiovasc. Magn. Reson. 14 (1), 83. 10.1186/1532-429X-14-83 23199362 PMC3552709

[B4] Barrett T. Wilhite S. E. Ledoux P. Evangelista C. Kim I. F. Tomashevsky M. (2013). NCBI GEO: Archive for Functional Genomics Data Sets-Update. Nucleic Acids Res. 41 (D1), D991–D995. 10.1093/nar/gks1193 23193258 PMC3531084

[B5] Chen Y. Chen W. Dai X. Zhang C. Zhang Q. Lu J. (2020). Identification of the Collagen Family as Prognostic Biomarkers and Immune-Associated Targets in Gastric Cancer. Int. Immunopharmacol. 87, 106798. 10.1016/j.intimp.2020.106798 32693357

[B6] Ding Y.-L. Sun S.-F. Zhao G.-L. (2021). COL5A2 as a Potential Clinical Biomarker for Gastric Cancer and Renal Metastasis. Medicine 100 (7), e24561. 10.1097/MD.0000000000024561 33607786 PMC7899835

[B7] Kalluri R. (2016). The Biology and Function of Fibroblasts in Cancer. Nat. Rev. Cancer 16, 582–598. 10.1038/nrc.2016.73 27550820

[B8] Koenig A. Mueller C. Hasel C. Adler G. Menke A. (2006). Collagen Type I Induces Disruption of E-Cadherin-Mediated Cell-Cell Contacts and Promotes Proliferation of Pancreatic Carcinoma Cells. Cancer Res. 66 (9), 4662–4671. 10.1158/0008-5472.CAN-05-2804 16651417

[B9] Li J. Ding Y. Li A. (2016). Identification of COL1A1 and COL1A2 as Candidate Prognostic Factors in Gastric Cancer. World J. Surg. Onc 14 (1), 297. 10.1186/s12957-016-1056-5 PMC5126984 27894325

[B10] Li L. Zhu Z. Zhao Y. Zhang Q. Wu X. Miao B. (2019). FN1, SPARC, and SERPINE1 are Highly Expressed and Significantly Related to a Poor Prognosis of Gastric Adenocarcinoma Revealed by Microarray and Bioinformatics. Sci. Rep. 9 (1), 7827. 10.1038/s41598-019-43924-x 31127138 PMC6534579

[B11] Li M. Wang J. Wang C. Xia L. Xu J. Xie X. (2020). Microenvironment Remodeled by Tumor and Stromal Cells Elevates Fibroblast-Derived COL1A1 and Facilitates Ovarian Cancer Metastasis. Exp. Cel Res. 394 (1), 112153. 10.1016/j.yexcr.2020.112153 32589888

[B12] Li Y. Sun R. Zhao X. Sun B. (2021). RUNX2 Promotes Malignant Progression in Gastric Cancer by Regulating COL1A1. Cancer Biomarkers 31 (3), 1–12. 10.3233/CBM-200472 33896817 PMC12500011

[B13] Li, Z. Z. LiLi A.-D. Xu L. Bai D.-W. Hou K.-Z. Zheng H.-C. (2016). SPARC Expression in Gastric Cancer Predicts Poor Prognosis: Results from a Clinical Cohort, Pooled Analysis and GSEA Assay. Oncotarget 7 (43), 70211–70222. 10.18632/oncotarget.12191 28053291 PMC5342547

[B14] Liao P. Li W. Liu R. Teer J. K. Xu B. Zhang W. (2018). Genome-Scale Analysis Identifies SERPINE1 and SPARC as Diagnostic and Prognostic Biomarkers in Gastric Cancer. OncoTargets Ther. 11, 6969–6980. 10.2147/OTT.S173934 PMC6199229 30410354

[B15] Liu J. Lichtenberg T. Hoadley K. A. Poisson L. M. Lazar A. J. Cherniack A. D. (2018). An Integrated TCGA Pan-Cancer Clinical Data Resource to Drive High-Quality Survival Outcome Analytics. Cell 173 (2), 400–e11. 10.1016/j.cell.2018.02.052 29625055 PMC6066282

[B16] Ma H.-P. Chang H.-L. Bamodu O. A. Yadav V. K. Huang T.-Y. Wu A. T. H. (2019). Collagen 1A1 (COL1A1) Is a Reliable Biomarker and Putative Therapeutic Target for Hepatocellular Carcinogenesis and Metastasis. Cancers 11 (6), 786. 10.3390/cancers11060786 PMC6627889 31181620

[B17] Madsen C. D. Sidenius. N. (2008). The Interaction between Urokinase Receptor and Vitronectin in Cell Adhesion and Signalling. Eur. J. Cell Biol. 87, 617–629. 10.1016/j.ejcb.2008.02.003 18353489

[B18] Marsico G. Russo L. Quondamatteo F. Pandit A. (2018). Glycosylation and Integrin Regulation in Cancer. Trends Cancer 4, 537–552. 10.1016/j.trecan.2018.05.009 30064662

[B19] Mohan V. Das A. Sagi I. (2020). Emerging Roles of ECM Remodeling Processes in Cancer. Semin. Cancer Biol. 62, 192–200. 10.1016/j.semcancer.2019.09.004 31518697

[B20] Najafi M. Farhood B. Mortezaee K. (2019). Extracellular Matrix (ECM) Stiffness and Degradation as Cancer Drivers. J. Cel Biochem 120 (3), 2782–2790. 10.1002/jcb.27681 30321449

[B21] Otasek D. Morris J. H. Bouças J. Pico A. R. Demchak B. (2019). Cytoscape Automation: Empowering Workflow-Based Network Analysis. Genome Biol. 20 (1), 185. 10.1186/s13059-019-1758-4 31477170 PMC6717989

[B22] Piersma B. Hayward M. K. Weaver V. M. (2020). Fibrosis and Cancer: A Strained Relationship. Biochim. Biophys. Acta (Bba) - Rev. Cancer 1873, 188356. 10.1016/j.bbcan.2020.188356 PMC7733542 32147542

[B23] Quante M. Tu S. P. Tomita H. Gonda T. Wang S. S. W. Takashi S. (2011). Bone Marrow-Derived Myofibroblasts Contribute to the Mesenchymal Stem Cell Niche and Promote Tumor Growth. Cancer Cell 19 (2), 257–272. 10.1016/j.ccr.2011.01.020 21316604 PMC3060401

[B24] Ren X. Chen X. Fang K. Zhang X. Wei X. Zhang T. (2021). COL5A2 Promotes Proliferation and Invasion in Prostate Cancer and is One of Seven Gleason-Related Genes that Predict Recurrence-Free Survival. Front. Oncol. 11, 583083. 10.3389/fonc.2021.583083 33816226 PMC8012814

[B25] Sato T. Oshima T. Yamamoto N. Yamada T. Hasegawa S. Yukawa N. (2013). Clinical Significance of SPARC Gene Expression in Patients with Gastric Cancer. J. Surg. Oncol. 108 (6), 364–368. 10.1002/jso.23425 24018911

[B26] Shi R. Gao S. Zhang J. Xu J. Graham L. M. Yang X. (2021). Collagen Prolyl 4-Hydroxylases Modify Tumor Progression. Acta Biochim. Biophys. Sinica 53, 805–814. 10.1093/abbs/gmab065 PMC8445723 34009234

[B27] Shi S. Zhang Z. G. (2019). Role of Sp1 Expression in Gastric Cancer: A Meta-Analysis and Bioinformatics Analysis. Oncol. Lett. 18 (4), 4126–4135. 10.3892/ol.2019.10775 31579418 PMC6757306

[B28] Shintani Y. Maeda M. Chaika N. Johnson K. R. Wheelock M. J. (2008). Collagen I Promotes Epithelial-to-Mesenchymal Transition in Lung Cancer Cells via Transforming Growth Factor-β Signaling. Am. J. Respir. Cel Mol Biol 38 (1), 95–104. 10.1165/rcmb.2007-0071OC PMC2176131 17673689

[B29] Song H. Liu L. Song Z. Ren Y. Li C. Huo J. (2018). P4HA3is Epigenetically Activated by Slug in Gastric Cancer and its Deregulation is Associated with Enhanced Metastasis and Poor Survival. Technol. Cancer Res. Treat. 17, 153303381879648. 10.1177/1533033818796485 PMC6131293 30198421

[B30] Sun C. Yuan Q. Wu D. Meng X. Wang B. (2017). Identification of Core Genes and Outcome in Gastric Cancer Using Bioinformatics Analysis. Oncotarget 8 (41), 70271–70280. 10.18632/oncotarget.20082 29050278 PMC5642553

[B31] Sung H. Ferlay J. Siegel R. L. Laversanne M. Soerjomataram I. Jemal A. (2021). Global Cancer Statistics 2020: GLOBOCAN Estimates of Incidence and Mortality Worldwide for 36 Cancers in 185 Countries. CA A. Cancer J. Clin. 71 (3), 209–249. 10.3322/caac.21660 33538338

[B32] Szklarczyk D. Gable A. L. Lyon D. Junge A. Wyder S. Huerta-Cepas J. (2019). STRING V11: Protein-Protein Association Networks with Increased Coverage, Supporting Functional Discovery in Genome-Wide Experimental Datasets. Nucleic Acids Res. 47 (D1), D607–D613. 10.1093/nar/gky1131 30476243 PMC6323986

[B33] Tai I. T. Tang M. J. (2008). SPARC in Cancer Biology: Its Role in Cancer Progression and Potential for Therapy. Drug Resist. Updates 11 (6), 231–246. 10.1016/j.drup.2008.08.005 18849185

[B34] Tan Y. Chen Q. Xing Y. Zhang C. Pan S. An W. (2021). High Expression of COL5A2, a Member of COL5 Family, Indicates the Poor Survival and Facilitates Cell Migration in Gastric Cancer. Biosci. Rep. 41 (4), BSR20204293. 10.1042/BSR20204293 33739392 PMC8039095

[B35] Tang Z. Li C. Kang B. Gao G. Li C. Zhang Z. (2017). GEPIA: A Web Server for Cancer and Normal Gene Expression Profiling and Interactive Analyses. Nucleic Acids Res. 45 (W1), W98–W102. 10.1093/nar/gkx247 28407145 PMC5570223

[B36] Vivian J. Rao A. A. Nothaft F. A. Ketchum C. Armstrong J. Novak A. (2017). Toil Enables Reproducible, Open Source, Big Biomedical Data Analyses. Nat. Biotechnol. 35, 314–316. 10.1038/nbt.3772 28398314 PMC5546205

[B37] Wang J. Jiang Y.-H. YangYang P.-Y. Liu F. (2021). Increased Collagen Type V α2 (COL5A2) in Colorectal Cancer Is Associated with Poor Prognosis and Tumor Progression. OncoTargets Ther. 14, 2991–3002. 10.2147/OTT.S288422 PMC8107053 33981148

[B38] Wang, L. L. Yang M. Shan L. Qi L. Chai C. Zhou Q. (2012). The Role of SPARC Protein Expression in the Progress of Gastric Cancer. Pathol. Oncol. Res. 18 (3), 697–702. 10.1007/s12253-012-9497-9 22246794 PMC3342504

[B39] Wang Q. Wen Y.-G. Li D.-P. Xia J. Zhou C.-Z. Yan D.-W. (2012). Upregulated INHBA Expression is Associated with Poor Survival in Gastric Cancer. Med. Oncol. 29 (1), 77–83. 10.1007/s12032-010-9766-y 21132402

[B40] Wang T. Wang Y.-X. Dong Y.-Q. Yu Y.-L. Ma K. (2020). Prolyl 4-Hydroxylase Subunit Alpha 3 Presents a Cancer Promotive Function in Head and Neck Squamous Cell Carcinoma via Regulating Epithelial-Mesenchymal Transition. Arch. Oral Biol. 113, 104711. 10.1016/j.archoralbio.2020.104711 32220804

[B41] Wang W. He Y. Zhao Q. Zhao X. Li Z. (2020). Identification of Potential Key Genes in Gastric Cancer Using Bioinformatics Analysis. Biom Rep. 12 (4), 178–192. 10.3892/br.2020.1281 PMC7054703 32190306

[B42] Wang Y. Zheng K. Chen X. Chen R. Zou Y. (2021). Bioinformatics Analysis Identifies COL1A1, THBS2 and SPP1 as Potential Predictors of Patient Prognosis and Immunotherapy Response in Gastric Cancer. Biosci. Rep. 41 (1), BSR20202564. 10.1042/BSR20202564 33345281 PMC7796188

[B43] Wang Z. Hao B. Yang Y. Wang R. Li Y. Wu Q. (2014). Prognostic Role of SPARC Expression in Gastric Cancer: A Meta-Analysis. Arch. Med. Sci. 10 (5), 863–869. 10.5114/aoms.2014.46207 25395936 PMC4223132

[B44] Yamazaki S. Higuchi Y. Ishibashi M. Hashimoto H. Yasunaga M. Matsumura Y. (2018). Collagen Type I Induces EGFR-TKI Resistance in EGFR-Mutated Cancer Cells by MTOR Activation through Akt-Independent Pathway. Cancer Sci. 109 (6), 2063–2073. 10.1111/cas.13624 29701925 PMC5989854

[B45] Yang M. C. Wang C. J. Liao P. C. Yen C. J. Shan Y. S. (2014). Hepatic Stellate Cells Secretes Type I Collagen to Trigger Epithelial Mesenchymal Transition of Hepatoma Cells. Am. J. Cancer Res. 4 (6), 751–763. 25520865 PMC4266709

[B46] Yu G. Wang L.-G. Han Y. HeHe Q.-Y. (2012). ClusterProfiler: An R Package for Comparing Biological Themes Among Gene Clusters. OMICS: A J. Integr. Biol. 16 (5), 284–287. 10.1089/omi.2011.0118 PMC3339379 22455463

[B47] Zhang J.-L. ChenChen G.-W. LiuLiu Y.-C. WangWang P.-Y. Wang X. WanWan Y.-L. (2012). Secreted Protein Acidic and Rich in Cysteine (SPARC) Suppresses Angiogenesis by Down-Regulating the Expression of VEGF and MMP-7 in Gastric Cancer. PLoS ONE 7 (9), e44618. 10.1371/journal.pone.0044618 22957090 PMC3434168

[B48] Zhang J. Wang P. Zhu J. Wang W. Yin J. Zhang C. (2014). SPARC Expression is Negatively Correlated with Clinicopathological Factors of Gastric Cancer and Inhibits Malignancy of Gastric Cancer Cells. Oncol. Rep. 31 (5), 2312–2320. 10.3892/or.2014.3118 24676680

[B49] Zhang Z. Wang Y. Zhang J. Zhong J. Yang R. (2018). COL1A1 Promotes Metastasis in Colorectal Cancer by Regulating the WNT/PCP Pathway. Mol. Med. Rep. 17 (4), 5037–5042. 10.3892/mmr.2018.8533 29393423 PMC5865965

[B50] Zhao Q. Xie J. Xie J. Zhao R. Song C. Wang H. (2021). Weighted Correlation Network Analysis Identifies FN1, COL1A1 and SERPINE1 Associated with the Progression and Prognosis of Gastric Cancer. Cancer Biomarkers 31 (1), 59–75. 10.3233/CBM-200594 33780362 PMC12499999

[B51] Zhao Z.-S. WangWang Y.-Y. Chu Y.-Q. Ye Z.-Y. TaoTao H.-Q. (2010). SPARC is Associated with Gastric Cancer Progression and Poor Survival of Patients. Clin. Cancer Res. 16 (1), 260–268. 10.1158/1078-0432.CCR-09-1247 20028745

[B52] Zheng H.-C. Gong B.-C. Zhao S. (2017). The Meta and Bioinformatics Analysis of GRP78 Expression in Gastric Cancer. Oncotarget 8 (42), 73017–73028. 10.18632/oncotarget.20318 29069845 PMC5641188

[B53] Zheng X. Liu W. Xiang J. Liu P. Ke M. Wang B. (2017). Collagen I Promotes Hepatocellular Carcinoma Cell Proliferation by Regulating Integrin β1/FAK Signaling Pathway in Nonalcoholic Fatty Liver. Oncotarget 8 (56), 95586–95595. 10.18632/oncotarget.21525 29221151 PMC5707045

